# Female Adult *Aedes albopictus* Suppression by *Wolbachia*-Infected Male Mosquitoes

**DOI:** 10.1038/srep33846

**Published:** 2016-09-23

**Authors:** James W. Mains, Corey L. Brelsfoard, Robert I. Rose, Stephen L. Dobson

**Affiliations:** 1 MosquitoMate, Inc., 2520 Regency Rd., Lexington, KY 40503, USA; 2 Biotechnology Regulatory Consultant, 8322 Sharon Dr., Frederick, MD 21704, USA; 3 Department of Entomology, University of Kentucky, Lexington, KY 40546, USA.

## Abstract

Dengue, chikungunya and zika viruses are pathogens with an increasing global impact. In the absence of an approved vaccine or therapy, their management relies on controlling the mosquito vectors. But traditional controls are inadequate, and the range of invasive species such as *Aedes albopictus* (Asian Tiger Mosquito) is expanding. Genetically modified mosquitoes are being tested, but their use has encountered regulatory barriers and public opposition in some countries. *Wolbachia* bacteria can cause a form of conditional sterility, which can provide an alternative to genetic modification or irradiation. It is unknown however, whether openly released, artificially infected male *Ae. albopictus* can competitively mate and sterilize females at a level adequate to suppress a field population. Also, the unintended establishment of *Wolbachia* at the introduction site could result from horizontal transmission or inadvertent female release. In 2014, an Experimental Use Permit from the United States Environmental Protection Agency approved a pilot field trial in Lexington, Kentucky, USA. Here, we present data showing localized reduction of both egg hatch and adult female numbers. The artificial *Wolbachia* type was not observed to establish in the field. The results are discussed in relation to the applied use of *Wolbachia*-infected males as a biopesticide to suppress field populations of *Ae. albopictus*.

Because of its success with many agricultural pests, there has been substantial effort invested in developing Sterile Insect Technique (SIT) to control medically important mosquito species. However, irradiation damage affecting mosquito fitness can impact efficacy. Maternally inherited *Wolbachia* bacteria can cause a form of conditional sterility known as Cytoplasmic Incompatibility (CI) in many insect species, including mosquitoes^1^. Contained trials show fewer negative fitness effects associated with some *Wolbachia*-infected strains, relative to radiation-induced sterility^2^. A prior field trial demonstrated that cytoplasmic incompatibility resulting from inundative male releases could suppress a population of *Culex* mosquitoes^3^. However, the *Culex* demonstration was constrained by an inability to manipulate *Wolbachia* infections and construct artificial *Wolbachia* infection types, and therefore the approach could not be extended to additional *Culex* populations or other important mosquito species.

Following invention of a method to generate artificial *Wolbachia* infection types^4^, much of the effort devoted to developing applied *Wolbachia*-based approaches has focused on a method known as population replacement, in which the introduction of *Wolbachia*-infected individuals are intended to establish the artificial *Wolbachia* infection type within the targeted mosquito population^5^,^6^. This can be pursued if the *Wolbachia* has desirable traits in the targeted population, such as *Wolbachia* interference with pathogen transmission^7^. While there are multiple examples of successful *Wolbachia* establishment, there are additional examples in which population replacement has not resulted^8^. Because *Wolbachia* is maternally inherited, population replacement approaches require the introduction of *Wolbachia*-infected females. Because female mosquitoes can bite, blood feed and transmit pathogens, there are ethical considerations, and biosafety regulations can restrict open release of female mosquitoes^9^,^10^. In contrast, male mosquitoes do not bite or blood feed, and repeated, inundative releases of male mosquitoes is the foundation of SIT-type approaches^7^.

We have previously developed strains of *Ae. albopictus* that are infected artificially with different *Wolbachia* types^11^. The *w*Pip infection type from *Culex pipiens* displays desirable characteristics for an applied population suppression approach, including high maternal inheritance rates, little measurable adverse effects on adult male competitiveness, and high cytoplasmic incompatibility levels^2^. Because laboratory tests are not necessarily predictive of outcomes in the field, we began working with regulatory authorities in the USA to obtain permits for open release field trials and began characterizing a field site in Lexington, Kentucky, USA as a potential test site. This location is typical suburban *Ae. albopictus* habitat and was selected due to its close proximity to the rearing facility and its robust *Ae. albopictus* population^12^.

To provide a ‘before/after comparison,’ the field sites were monitored in 2013, prior to the introduction of incompatible males, using BG traps and ovitraps. The goals were to characterize *Ae. albopictus* egg hatch rate, adult female and adult male number, with the intention to compare the resulting data with similar measurements made the subsequent year, during the incompatible male introductions. Furthermore, the 2013 field data served as the basis for selecting the site for the 2014 intervention. The 2013 results were grouped, analyzed and discussed in terms of the resulting incompatible male introduction and non-introduction areas. These were subsequently referred to as the Treated and Untreated areas, respectively. Importantly, the Treated area did not receive incompatible males or any other *Ae. albopictus* abatement measures in 2013. But to simplify and facilitate comparisons, we refer to the areas using the same terminology as 2014.

The overall number of adult female *Ae. albopictus* collected in 2013 did not differ between the Treated and Untreated areas, and a similar pattern of seasonal population increase and decline was observed at both areas (Fig. 1a). Relatively few females were collected early in the season, *i.e*., June collections accounted for few of the total females collected for the year. Approximately a third of the total collected females were from July and an additional third in August. The population size declined in September as the weather became cooler, drier and the population went into diapause, and by October, few *Ae. albopictus* were collected.

Fewer *Ae. albopictus* males were collected in BG traps (Fig. S1), relative to the females caught in the same traps. A lower capture rate of males with BG traps is consistent with prior reports^13^,^14^. For both areas, the percent sex ratio of BG collected adults averaged 79 ± 15% (Mean ± Std Dev). The seasonal pattern of males was similar to that described for females above. No difference was observed in the male number, comparing between the Treated and Untreated areas for each month (*p* > 0.17).

Ovitraps were placed in May 2013, and the earliest eggs were observed in June. The percent egg hatch remained at approximately 65% at both areas through August and was subsequently observed to decline in September (*p* < 0.0001), as eggs at both areas began to enter into diapause (Fig. 1b). Comparing between the two areas for each month, no statistical difference was observed in egg hatch (*p* > 0.21), and similar numbers of eggs were collected between the two areas (data not shown).

In 2014, after receiving an Experimental Use Permit from the USA Environmental Protection Agency (89668-EUP-1) for the open release of *Ae. albopictus* males infected with the *w*Pip *Wolbachia* type, field collections of eggs and adults were started in June. At the Untreated area, the overall seasonal pattern was similar to that in the preceding year. Relatively few females were collected in June, followed by a population size increase in July and August (Fig. 2a). The overall population density remained high in September at the Untreated area. With the onset of cooler weather in October, the population declined and few additional adults were collected. The pattern of males was similar to that of 2013, with fewer adult males collected in BG traps, relative to females collected in the same traps (Fig. S2). The overall percent sex ratio of BG collected adults in the Untreated area was 80 ± 12% (Mean ± Std Dev).

At the Treated area, incompatible male introductions began in June 2014 with approximately 5,000 males introduced twice a week, for a total of 10,000 males per week. Males were released from a single location. Incompatible male introductions continued through September, for a total of 17 weeks. In total, 182,000 incompatible males were introduced into the Treated area. BG trap collections showed more males collected within the Treated area in June (*χ*^2^(1,N = 89) = 12.301, *p* < 0.0005) and July (*χ*^2^(1,N = 108) = 11.959, *p* < 0.0005), compared to the Untreated area (Fig. S2). Within the Treated area, the number of collected adult males and the sex ratio of collected adults were both correlated with distance from the male introduction point (Fig. S3). This is consistent with expectations for a point release of incompatible males, which are dispersing away from the release point over time.

Early season comparison between the Treated and Untreated areas show no difference in female number in June and July (Fig. 2a). During the peak months for mosquito density, fewer *Ae. albopictus* females were collected at the Treated area in August (*χ*^2^(1,N = 97) = 7.012, *p* < 0.0081) and September (*χ*^2^(1,N = 104) = 10.309, *p* < 0.0013), compared to collections in the Untreated area during the same periods. BG trap collections within the Treated area did not show a correlation between adult female number and distance from the male introduction point (*p* > 0.13).

Cytoplasmic incompatibility is characterized by early developmental arrest of embryos. Therefore, if the difference in adult female number between the Treated and Untreated areas were due to cytoplasmic incompatibility, a reduction in egg hatch would be expected. The egg hatch at the Treated area was lower (Fig. 2b) than that observed in the Untreated area in June (*χ*^2^(1,N = 146) = 13.087, *p* < 0.0003), July (*χ*^2^(1,N = 209) = 44.917, *p* < 0.0001) and August (*χ*^2^(1,N = 200) = 51.0174, *p* < 0.0001). In September, the egg hatch declined at both areas, which is consistent with expectations for the onset of diapause. Similar numbers of eggs were collected at the Untreated and Treated sites (data not shown).

Consistent with expectations for an effect caused by incompatible matings between wild-type females and introduced males, eggs that were collected further from the male introduction point experienced higher egg hatch rates (Fig. 3). Significant correlations between egg hatch and distance from the male introduction point were observed in July and August. Similar but non-significant trends were observed in June and September (Fig. 3). The latter may reflect complicating factors of egg diapause, *i.e*., lower egg hatch results during short day-length periods in the spring and fall (Fig. 2). Conducting a similar analysis on the 2013 data, examining for a decline in hatch rate across the same trap locations (*i.e*., prior to incompatible male releases) did not detect a correlation between hatch rate and location.

*Wolbachia* is maternally inherited. Therefore the unintentional introduction of *w*Pip infected females could lead to the establishment of the *w*Pip infection type in the targeted *Ae. albopictus* population. To monitor for *w*Pip establishment, a subset of eggs collected from the Treated area were hatched, reared to adult, and assessed for *Wolbachia* type using PCR. PCR assays of resulting female adults were repeated twice a month. The following spring, additional PCR assays were conducted with eggs collected in May 2015. None of the PCR tested individuals were infected with the *w*Pip *Wolbachia* type (n = 61).

To examine for a potential effect of inundative releases on non-targeted mosquitoes occurring at the Treated site, non-*Ae. albopictus* mosquitoes collected in BG traps were also monitored. Other than *Ae. albopictus*, relatively few additional mosquito species were collected using the BG traps (Fig. S4). A majority of these were *Culex* spp, with a total of 105 females and 67 males collected between June and September. Additional species totaled 30 females and 2 males, most of which were *Aedes triseriatus*, a native mosquito that often co-occurs with *Aedes albopictus*^14^. Sporadic, rare collections of *Toxorhychites rutilus*, *Orthopodmyia signifiera*, *Anopheles quadrimaculatus*, *Aedes japonicus* and *Aedes trivittatus* were also identified. Comparing between the Treated and Untreated areas, no difference was observed in the number of non-target mosquito species that were collected.

Weather patterns are important drivers of mosquito population dynamics. Comparing the weather conditions between years, the 2014 field season was generally warmer than 2013 (Fig. S5). Furthermore, 2013 was wetter early in the season, relative to early season 2014. In contrast, more rainfall occurred late in the 2014 season, compared to the 2013 late season. Taken together, the late season of 2014 was both wetter and warmer than 2013, which is correlated to a generally larger mosquito population in 2014, relative to 2013. Specifically, in the Untreated area, more females were observed in the late season 2014, compared to late season 2013.

Based on prior mark release recapture studies of *Ae. albopictus* male dispersal in urban environments^15^, we estimate the Treatment area to encompass ~12.5 ha, which gives an introduction rate of ~800 incompatible males/ha/week, which is a relatively modest introduction rate compared to prior studies, *e.g.*, 14,000–100,000/ha/wk^16^,^17^.

The study site is notable also in that it is not isolated from immigrating, indigenous *Ae. albopictus*. Prior MRR studies estimate female *Ae. albopictus* to travel up to 290 m over their lifetime^18^. Therefore *Ae. albopictus* females originating outside the Treatment area can readily move into and across the area. Furthermore, females that mate with incompatible males inside the Treated area can exit the area, diluting the ability to detect an effect. Female dispersal may have contributed to the observed lack of correlation between adult female number and distance from the male introduction point.

The results demonstrate a significant reduction in the overall number of adult females within the Treatment area, compared to that of the Untreated area. This result is notable, given the relatively small size of the treatment area and the introduction of incompatible males from a single point. A dogma for SIT-type studies is that the approach must be ‘area wide,’ *i.e*., at a scale exceeding the normal flight range of the targeted insect. Or alternatively, trials are often conducted on ecological or physical islands, where the population is insulated from immigration^16^,^17^,^19^. As described above, small scale tests in which the population is not isolated can be complicated by ‘edge effects,’ *e.g*., mated, indigenous females that immigrate into the area are less likely to be affected by the incompatible males, because females tend towards monogamy^20^. The results shown here suggest an additional method in which point releases of incompatible males cause localized reductions of adult *Ae. albopictus* females.

For experimental purposes, the *Wolbachia* pesticide method has been used in isolation here. But it is emphasized that the downstream integration of the *Wolbachia* approach with additional control methods may improve overall cost and efficiency. For example, integration with chemical or biological larvicides would not be anticipated to negatively affect the introduced incompatible males, and because the impact of each incompatible male is greater when there are fewer competing males, population reduction caused by a larvicide may have a synergistic effect.

In conclusion, the results suggest the feasibility of *Wolbachia* as a pesticide against *Ae. albopictus* and encourage additional work examining the approach within different ecological contexts and at a larger scale. While entomological endpoints made up the present study, future large-scale work within disease endemic areas can allow measuring for an effect on pathogen transmission. Because *Wolbachia* is known to cause cytoplasmic incompatibility in other mosquitoes, similar field trials might be conducted with additional medically important species, *e.g*., *Ae. aegypti* and *Culex pipiens*.

## Methods

### Mosquito stocks, egg hatch, rearing and introduction

The methods used to construct the artificially-infected *Ae. albopictus* strain have been described previously^11^,^21^. In brief, the *w*Pip *Wolbachia* infection was microinjected into an aposymbiotic strain of *Ae. albopictus*. Laboratory crosses of the resulting strain show *w*Pip males to be competitive and fully incompatible with naturally-infected *Ae. albopictus* females^2^,^22^.

Colony rearing was at 29 °C, 73% RH and a photoperiod of 16:8 light:dark. Larvae were reared in pans (Pactive, Lake Forest, IL) containing 500 ml of filtered water and fed liver powder (MP Biomedicals LLC, Solon, OH) *ad libitum*. Adults were provided a constant supply of 10% sucrose and held in 24.5 cm^3^ cages (MegaView Science Co., Taichung, Taiwan). Adults in all experiments were blood fed using sausage casing stretched over a 20 ml plastic cup (Pactive, Lake Forest, IL), filled with approximately 10 ml of bovine blood and heated to 37 °C.

Pupae were separated by sex using a mechanical separation device (John Hock Co., Gainsville, FL, USA). Pupae designated for releases were allowed to eclose in a cage, and the resulting adults were examined to remove any residual females.

The sites were selected in 2013. Homeowner voluntary agreement to participate in the study drove the initial site selection. The field sites are suburban area in Lexington, KY, USA. Lexington is located at the edge of the Cumberland Plateau with an average elevation of 294 m above sea level. Lexington is the second largest city in Kentucky, with a population density of 354 people per square kilometer. Lexington is in the northern periphery of a humid subtropical climate zone with four distinct seasons. While temperature extremes are unusual, Lexington is subject to rapid changes in temperature. Precipitation is fairly constant year round, with an average of 75 to 100 mm per month.

The Treated site is defined as a 250 m radius around a single release point [GPS: 38.022, −84.515]. Untreated sites are located near the release point (≤1.5 km), such that they were of similar habitat and experienced similar rainfall, temperature and humidity, but were located outside the typical flight range of *Ae. albopictus* mosquitoes^15^,^22^, *i.e*., greater than 250 m away from the release point. In 2013, using BG traps and ovicups, eight and six sites were monitored in the Untreated and Treated areas, respectively. In 2014, eleven and fifteen sites were monitored in the Untreated and Treated areas, respectively.

No incompatible male releases were performed in 2013. All sites were monitored in the absence of male introductions. Following receipt of the EPA permit, an initial release rate of 10,000 mosquitoes/week was targeted based on prior Mark Release Recapture studies that suggested that this rate would achieve a 10:1 ratio of Incompatible:Indigenous males (data not shown). Introduced males were divided into two releases of 5,000 per week, with the intent to better sustain numbers of active, incompatible males. Males were transported to the field in cardboard mailing tubes (2″ diameter; Art Wall Kraft #P2030K-6).

### Field Monitoring

The adult population was monitored using BG Sentinel traps with the BG lure (Biogent Sentinel, Regensburg, Germany). Traps were run for 24 hours once per week, and the collected mosquitoes were frozen and identified using a Leica EZ4D microscope (Leica Microsystems Inc, Buffalo Grove, IL, USA). Percent sex ratio is calculated as number of adult females divided by the total number of adults collected. Artificial ovisites used to monitor eggs consisted of a plastic cemetery vase (Reiss Innovations LLC, Manchester, CT, USA) lined with heavy seed germination paper (Anchor Paper Co., Saint Paul, MN USA) and containing approximately 100 ml of water. The paper within ovitraps was replaced weekly. Collected eggs were held for seven days for maturation, counted and then submerged to hatch. To improve the overall egg hatch and achieve hatch rates similar to that observed with lab reared mosquitoes, the methods used during the egg holding period were altered between the 2013 and 2014 field seasons. Specifically, in 2014 the papers were kept damp and within a humid plastic box, compared to 2013 when papers were allowed to dry on tabletops. With each year, egg papers from the Treated and Untreated sites were manipulated identically. Hatched and unhatched eggs were counted using the dissection scope. To examine for unintended establishment of the wPip infection at the field site, a subset of larvae hatching from collected eggs were reared to adults and PCR tested using the orf7A primer set. Weather data was downloaded from a local weather station (Weather Underground; wunderground.com).

### PCR

PCR was used to monitor *Wolbachia* infection type. Early in the season, PCR assays were performed once per three weeks. Later in the season, the frequency was increased to once per week. Adult mosquitoes were homogenized in 100 *μ*l of buffer containing 10 mM Tris-HCL, 1 mM EDTA, and 50 mM NaCl, at pH 8.2. After homogenization, samples were incubated at 100 °C five min and centrifuged at 16,000 g for five min. The *orf7A* (*orf7-Af*, GCTAATAGCACGAAATCGAAAC; *orf7-Ar*, AT T TCTCTAC-GACAGT TCTCC) primer set was used to diagnose the *w*Pip infection^23^. The general *Wolbachia* primers *WspecF*: 5′-CATACCTATTCGAAGGGATAG-3′ and *wspecR*: 5′-AGCTTCGAGTGAAACCAATTC-3′, were used to detect the naturally occurring *Wolbachia* type found in wild type *Aedes albopictus*^24^. 12S mitochondrial primers (*12SAI*-AAACTAGGATTAGATACCCTATTAT and *12SBI*-AAGAGCGACGGGCGATGTGT) were used to amplify mitochondria DNA as a positive control for template DNA quality^25^. *Culex pipiens* females (naturally infected with *w*Pip) were used for a positive control in PCR rxns. For all reactions, one *μ*l of the isolated DNA was amplified in 50 mM KCl, 20 mM Tris–HCl (pH 8.4), 1.5 mM MgCl_2_, 0.25 mM dNTPs, 0.5 mM primers, and 1 U Taq DNA polymerase in a total volume of 20 ml. Samples were denatured for three min at 94 °C, cycled 35 times at 94, 62, and 72 °C (1 min each), followed by a 10 min extension at 72 °C using a PTC-200 Thermal Cycler (Bio-Rad, Hercules, CA, USA). A volume of eight *μ*l of each amplification was separated on 1.5% agarose gels, stained with GelRed (Biotium, Hayward, CA, USA), and visualized under ultraviolet illumination.

### Statistical analyses

Statistical analyses were performed using JMP 12.2 and SAS 9.3 software (SAS Institute, Cary, NC). Non-parametric analysis was used (Wilcoxon) to compare within months and between areas in the number of adults and egg hatch rates. A Steel Dwass multiple comparison test was used to examine within area and across months. A bonferroni correction was used when multiple tests were compared.

## Additional Information

**How to cite this article**: Mains, J. W. *et al*. Female Adult *Aedes albopictus* Suppression by *Wolbachia*-Infected Male Mosquitoes. *Sci. Rep*. **6**, 33846; doi: 10.1038/srep33846 (2016).

## Supplementary Material

Supplementary Information

## Figures and Tables

**Figure 1:**
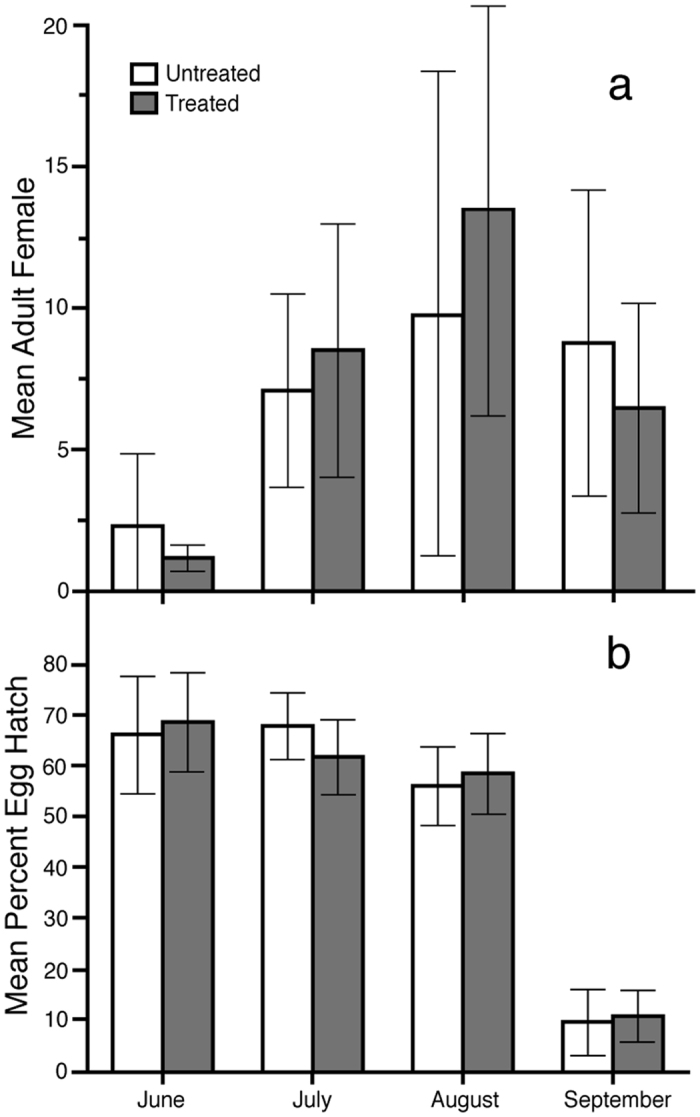
Mean (**a**) number of female adult *Ae. albopictus* collected using BG traps and (**b**) percent hatch of *Ae. albopictus* eggs collected using oviposition traps at the Treated and Untreated sites in the 2013 field season. There were no incompatible males introduced at either site in 2013. Bars show 95% confidence intervals.

**Figure 2:**
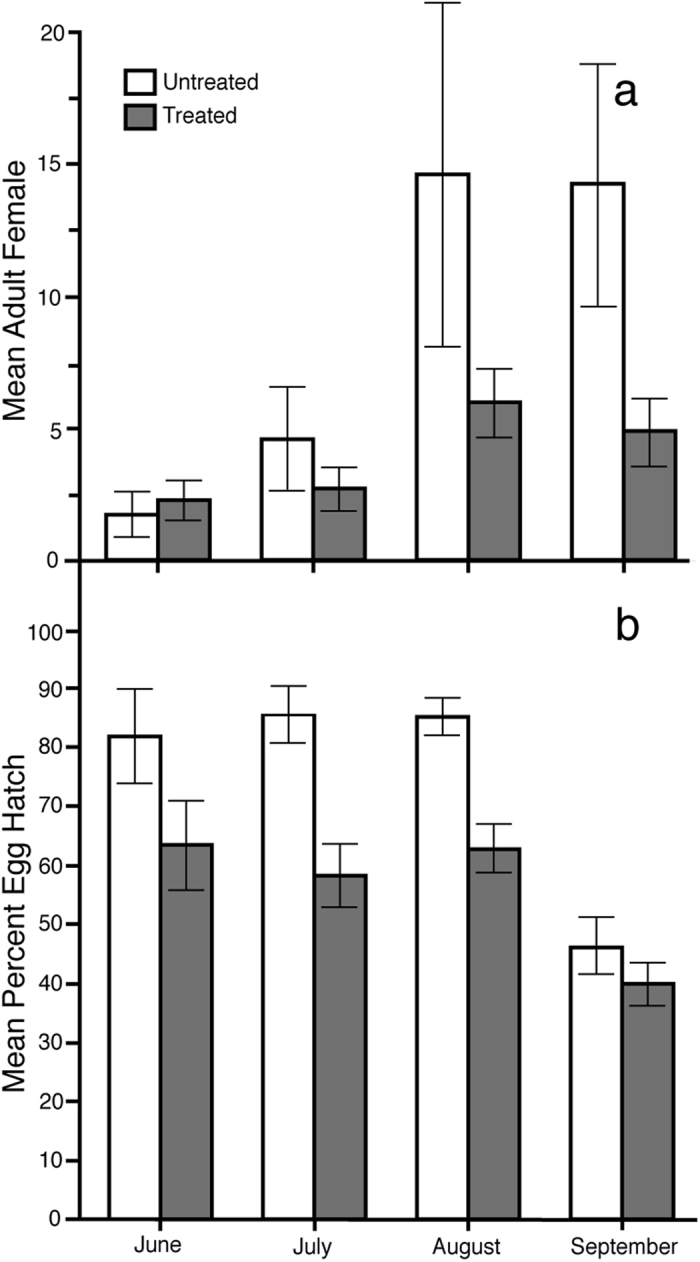
Mean (**a**) number of female adult *Ae. albopictus* collected using BG traps and (**b**) percent hatch of *Ae. albopictus* eggs collected using oviposition traps at the Treated and Untreated sites in the 2014 field season. In 2014, incompatible males were introduced at the Treated site only. Bars show 95% confidence intervals.

**Figure 3:**
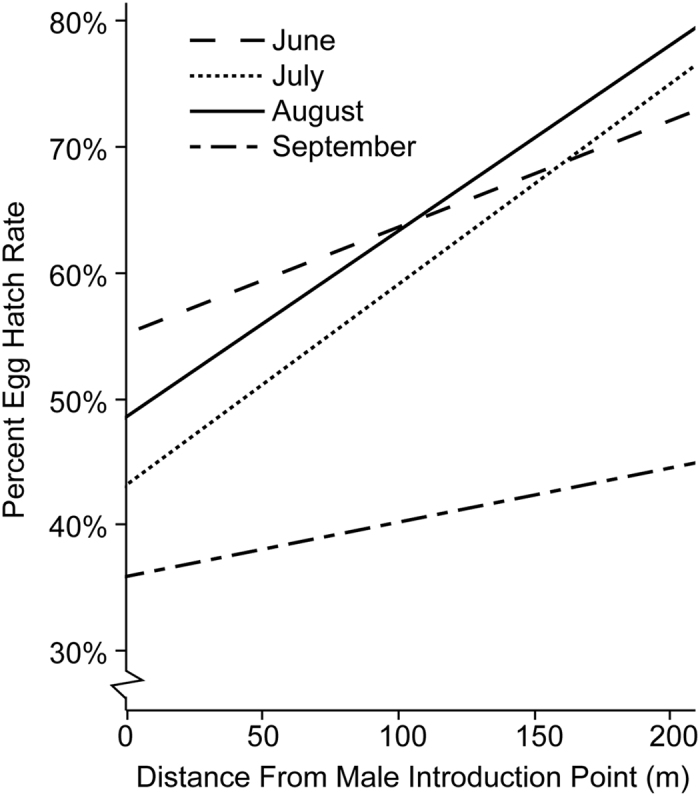
Egg hatch is correlated with distance from the male introduction point, among sites within the Treated area. Eggs that were collected closer to the male introduction point experienced a lower hatch rate in July (R^2^ = 0.105, F(1,129) = 14.96, *p* < 0.0002) and August (R^2^ = 0.158, F(1,118) = 21.94, *p* < 0.0001). Similar but non-significant trends were observed in June and September (*p* > 0.13).
