# Supplementary material for: Female Adult Aedes albopictus Suppression by Wolbachia-Infected Male Mosquitoes
Source: Sci Rep. 2016 Sep 23;6:33846. doi: 10.1038/srep33846 (PMC5034338; doi:10.1038/srep33846)
Supplement: Supplementary Information [file srep33846-s1.pdf]

SUPPORTING ONLINE MATERIAL

Female Adult *Aedes albopictus* Suppression by *Wolbachia*-Infected Male Mosquitoes

James W. Mains[1], Corey L. Brelsfoard[1], Robert I. Rose[2], and Stephen L. Dobson[1,3*]

[1] MosquitoMate, Inc., 2520 Regency Rd., Lexington, KY 40503

[2] Biotechnology Regulatory Consultant, 8322 Sharon Dr., Frederick, MD 21704

[3] Department of Entomology, University of Kentucky, Lexington, KY 40546

* To whom correspondence should be addressed: sdobson@mosquitomate.com

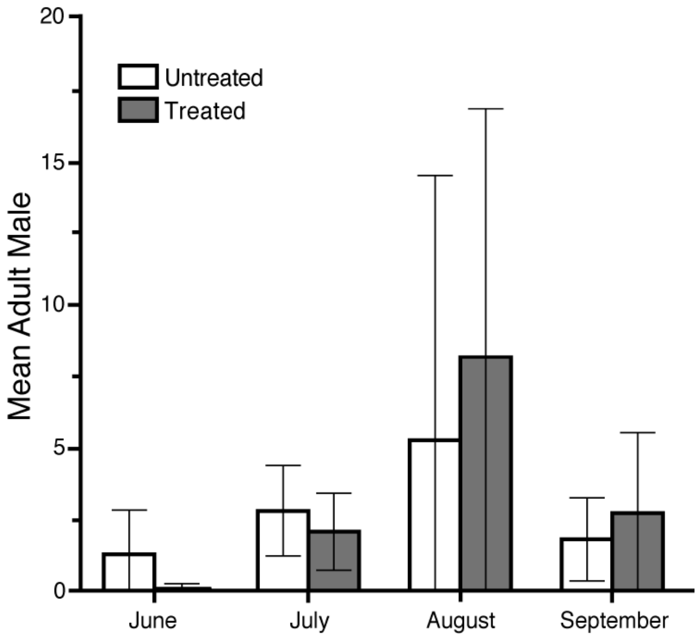

Figure S1. Mean number of male adult *Ae. albopictus* collected using BG traps at the Treated and Untreated sites in the 2013 field season. There were no incompatible males introduced at either site in 2013. Bars show 95% confidence intervals.

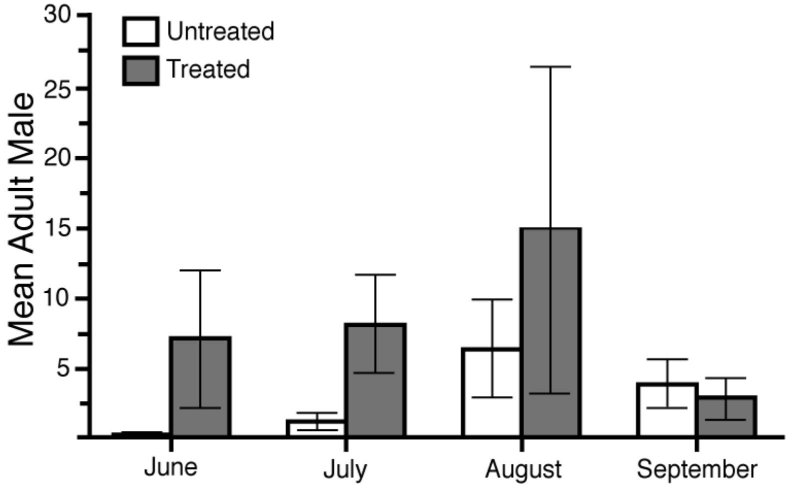

Figure S2. Mean number of male adult *Ae. albopictus* collected using BG traps at the Treated and Untreated sites in the 2014 field season. In 2014, incompatible males were introduced at the Treated site. Bars show 95% confidence intervals.

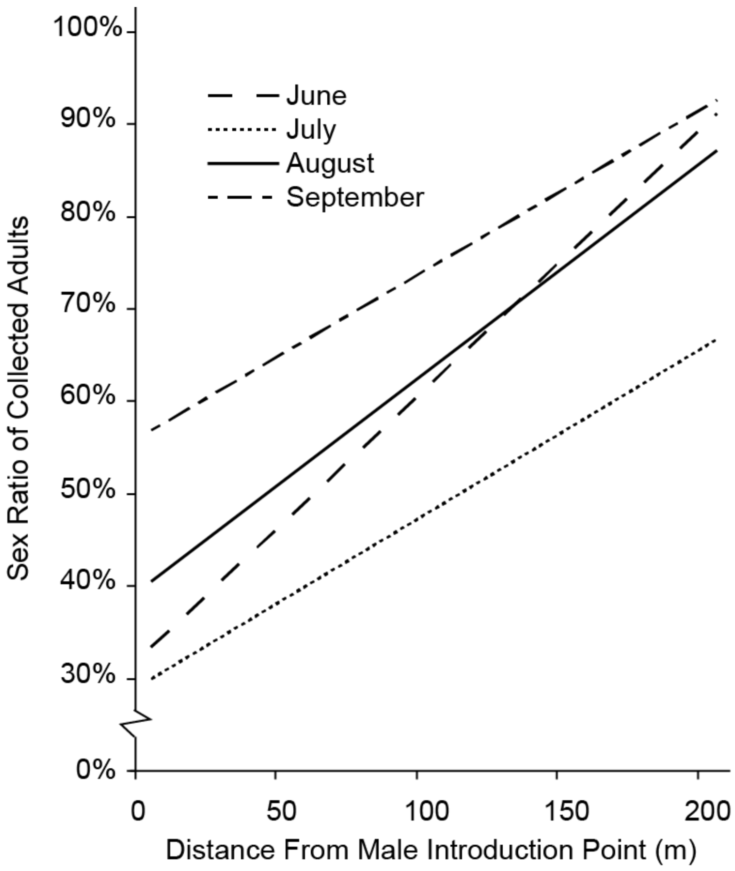

Figure S3. Within the Treatment area, the sex ratio of collected adults is correlated with distance from the male introduction point. Specifically, a lower Sex Ratio (*i.e.*, lower proportion of females) was observed in BG collections made near the male introduction point. Similar trends were observed in June ($R^2 = 0.198$, $F(1,43)=10.34$, $p < 0.0025$), July ($R^2 = 0.103$, $F(1,54)=6.124$, $p < 0.0166$), August ($R^2 = 0.193$, $F(1,54)=12.69$, $p < 0.0008$) and September ($R^2 = 0.124$, $F(1,57)=7.93$, $p < 0.0067$). Sex Ratio is defined as the number of adult females divided by the total number of adults (*i.e.*, adult males and females).

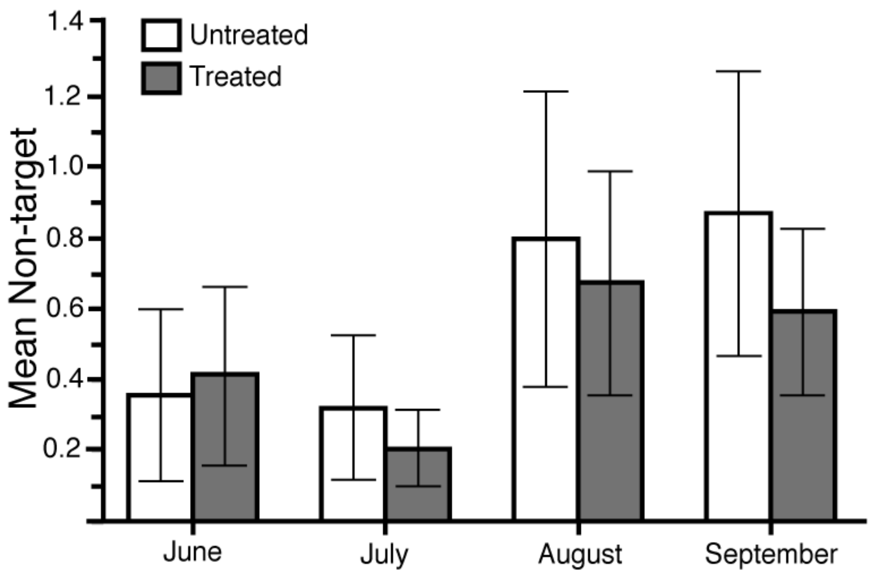

Figure S4. The mean number of non-*Ae. albopictus* mosquitoes, *i.e.*, non target mosquitoes, collected using BG traps at the Treated and Untreated sites in the 2014 field season. In 2014, incompatible males were introduced at the Treated site. Bars show 95% confidence intervals.

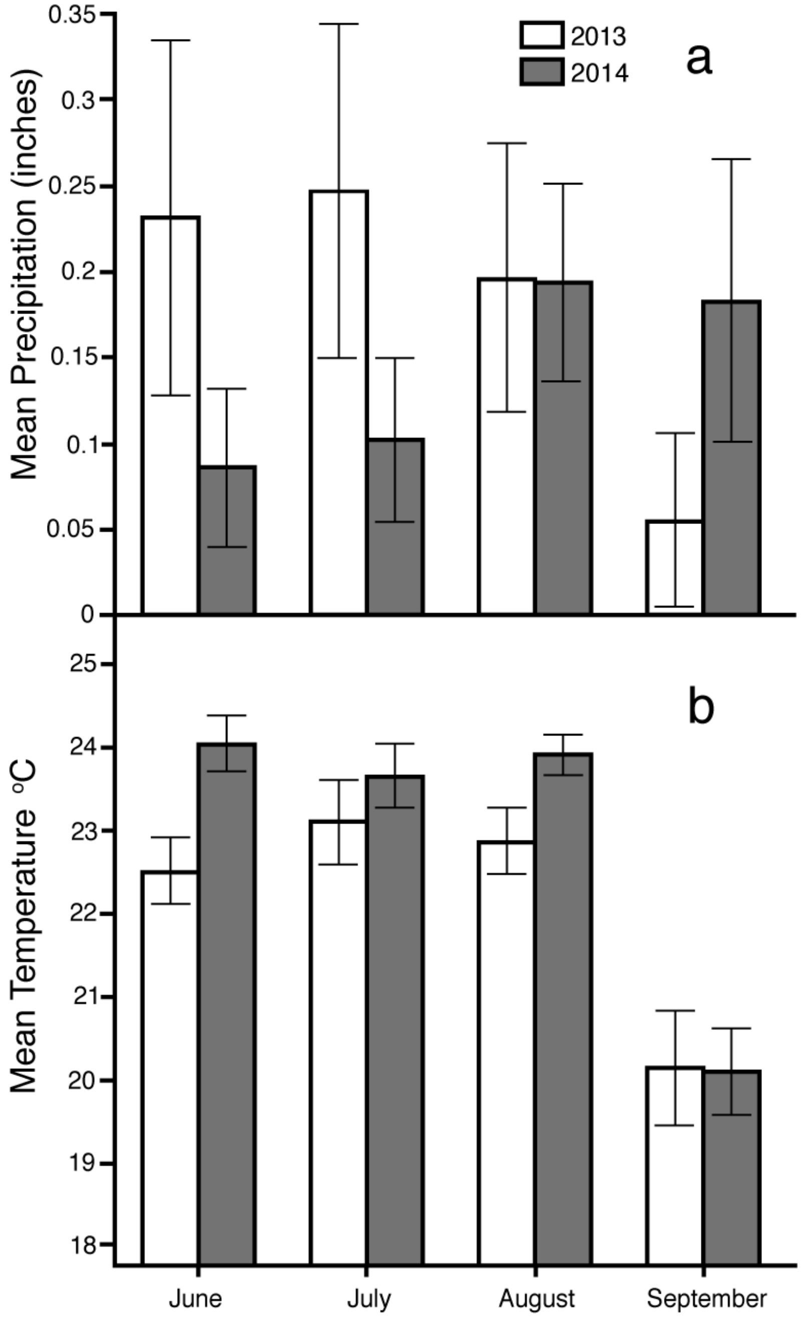

Figure S5. Mean daily (a) precipitation and (b) temperature during the monitoring periods of 2013 and 2014. Bars show standard error.
